# Design and Flight Experiment of a Motor-Directly-Driven Flapping-Wing Micro Air Vehicle with Extension Springs

**DOI:** 10.3390/biomimetics10100686

**Published:** 2025-10-12

**Authors:** Seungik Choi, Changyong Oh, Taesam Kang, Jungkeun Park

**Affiliations:** 1 Department of Smart Vehicle Engineering, Konkuk University, Seoul 05029, Republic of Korea; win2637@konkuk.ac.kr; 2 Department of Aerospace Information Engineering, Konkuk University, Seoul 05029, Republic of Korea; ocy4262@konkuk.ac.kr (C.O.); tskang@konkuk.ac.kr (T.K.)

**Keywords:** flapping-wing micro air vehicle, directly driven mechanism, extension spring, resonant frequency, PD control, attitude control

## Abstract

This study presents the design, control, and flight experiments of a motor-directly-driven flapping-wing micro air vehicle with extension springs (MDD-FWMAVES). The flapping wing actuation utilizes the resonance of a linear extension spring and a flapping wing. The analysis results of the proposed MDD-FWMAVES revealed a resonant frequency of 19.59 Hz for the flapping-wing mechanism, and actual flapping experiments confirmed this to be 20 Hz. Using a six-axis load cell, we demonstrated the ability to generate roll, pitch, and yaw moments for attitude control based on wing flapping variations. All roll, pitch, and yaw moments were linearly proportional to the wing flapping variations. MEMS gyroscopes and accelerometers were used to measure roll, pitch, and yaw angular velocities and the gravity. A complementary filter was applied to these measurements to obtain the roll and pitch angles required for attitude control. A microprocessor, two motor drive circuits, one MEMS gyroscope/accelerometer, and one EEPROM for flight data storage were implemented on a single, ultra-compact electronic control board and mounted on the MDD-FWMAVES. Simple roll and pitch PD controllers were implemented on this electronic control board, and the controlled flight feasibility of the MDD-FWMAVES was explored. Flight tests demonstrated stable hovering for approximately 6 s. While yaw control was not achieved, the onboard feedback control system demonstrated stable roll and pitch control. Therefore, the MDD-FWMAVES holds the potential to be developed into a high-performance flapping-wing micro air vehicle if its flight system and controller are improved.

## 1. Introduction

In recent decades, active research has been conducted on biomimetic flying vehicles that mimic the wing movements of insects and birds. Researchers study the morphology and wing flapping mechanisms of insects and birds and utilize these insights to develop flapping-wing micro air vehicles (FWMAVs). While both birds and insects generate lift by flapping their wings, their underlying aerodynamic force generation principles and control strategies are fundamentally different [1].

Most birds flap with a nearly vertical wing plane, producing lift and thrust primarily during the downstroke, and they employ their tail for stability and directional control. By combining tail control with wing shape modulation, birds achieve efficient flight in the intermediate Reynolds number regime [2,3].

In contrast, insects flap their wings at higher frequencies and larger amplitudes, generating lift and thrust during both the upstroke and the downstroke in a nearly horizontal plane. Insects exploit unsteady aerodynamic mechanisms, including leading edge vortex (LEV) stabilization, rotational circulation, wake capture, and clap and fling effects, to secure sufficient lift even in the low Reynolds number regime [4,5]. Moreover, lacking a tail, insects achieve roll, pitch, and yaw control solely through wing kinematics [6]. Although this tailless configuration provides less passive stability compared to tailed flyers, it enables hovering and agile maneuvering when supported by active feedback control [7].

Insect-inspired FWMAVs can achieve attitude control solely through wing movements and without a tail, demonstrating advantages such as high propulsion efficiency, hovering capability, and agile maneuverability [7,8]. Furthermore, insect-inspired FWMAVs can perform reconnaissance and detection missions in confined and complex spaces, and research is also underway on their potential for space flight [7,9].

Insect-inspired FWMAVs are implemented using various flapping drive systems, including motors, piezoelectric actuators, and electromagnetic actuators [7,10,11]. Among these, motor-based wing flapping has been the subject of extensive research and development in the areas of mechanical structure and flight control. Motor-based wing flapping mechanisms can be categorized into indirect and direct drive methods.

In the indirect drive systems, the unidirectional rotational motion of the motor is converted into flapping motion using a transmission mechanism. Since a single motor cannot independently control both wings, servo motors are used to control roll, pitch, and yaw [12,13,14,15,16,17]. The KU Beetle model successfully achieved flight by using a single motor to flap the wings and servo motors to control roll, pitch, and yaw [7,14,15,16]. In the other indirect drive systems, where separate motors are used for each wing, each wing can be controlled independently, allowing for roll control without an additional device. However, additional servo motors are still required to generate yaw and pitch torques [18,19,20,21].

Direct drive systems achieve wing flapping by directly connecting the motors to the wings, eliminating complex transmission mechanisms and additional servo motors. Direct drive systems require bidirectional motor operations, and the amplitude, position offset, and frequency of each wing can be individually controlled to generate roll, pitch, and yaw moments, as well as lift force for flight. However, reversing the driving direction during bidirectional drive requires significant energy consumption to overcome the large inertial forces. To address this issue, a flapping-wing system using a resonant mechanism has been proposed, which uses elastic materials to store inertial energy and then uses the stored energy when reversing the driving direction [22,23,24,25].

Helical or torsional springs have been primarily used as elastic materials for resonance [26,27,28,29,30,31,32]. A representative example is the flapping-wing hummingbird robot [31,32]. However, these springs are prone to fatigue failure due to large displacements and are difficult to align zero position during assembly [33].

To overcome these limitations, a motor-directly-driven flapping-wing micro air vehicle with extension springs (MDD-FWMAVES) was designed, and a prototype model was built and tested. It is reported that the roll, pitch, and yaw control torques can be generated directly using the prototype model [34]. Additionally, thrust tests were conducted using off-board motor drive modules and control boards. The experimental results showed that sufficient thrust can be generated for flight.

In this paper, we designed and implemented a flight controller for the insect-inspired MDD-FWMAVES that solves some of the critical problems of existing vehicles. First, large and uneven yaw moments were generated during wing flapping, making flight difficult. To solve this problem, the spring positions of the two wings, which were previously arranged asymmetrically, were changed to symmetrical ones. Second, wider wings were used to increase thrust and improve flight efficiency. Furthermore, the mass of each component was precisely measured using a high-resolution balance and incorporated into the design process, ensuring sufficient thrust margin relative to the reduced overall mass. Third, using a six-axis load cell, we characterized the lift force and roll, pitch, and yaw moments generated by the MDD-FWMAVES. The results revealed a linear dependence of the moments on control input, thereby confirming the feasibility of attitude control without a tail or auxiliary servomechanisms. Fourth, flight control, an IMU-based attitude estimation filter, and a proportional-differential (PD) controller were designed. The IMU filter reduced vibration noise generated during flight and enabled more stable and accurate attitude control. The performance was verified using a single-axis gimbal experiment, demonstrating effective vibration suppression and accurate attitude stabilization. Based on this result, a lightweight onboard control board integrating an MCU, IMU, and motor driver was developed to implement the proposed controller in real time. Finally, a hovering experiment was performed with the insect-inspired MDD-FWMAVES to demonstrate the attitude control performance of the onboard system.

## 2. Motor-Directly-Driven Flapping-Wing Micro Air Vehicle with Extension Springs

This section describes the design and basic operating principles of the MDD-FWMAVES. The dynamics and resonant frequency derivation of the flapping-wing system using extension springs are also briefly described.

### 2.1. Design of the MDD-FWMAVES

**Figure 1:**
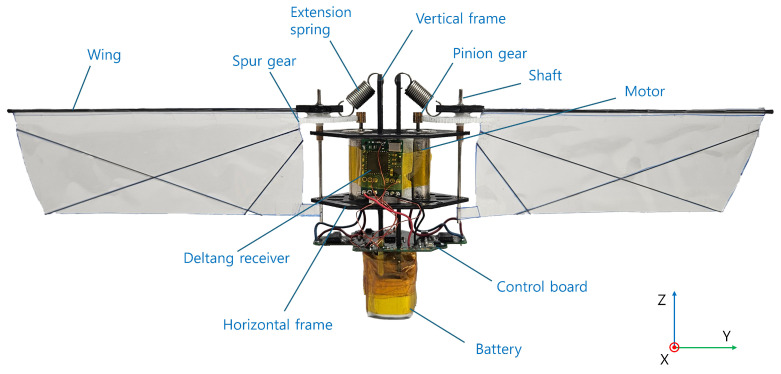
MDD-FWMAVES components and coordinate system.

Figure 1 shows the geometry and coordinate system of the MDD-FWMAVES developed in this study. The overall dimensions of the MDD-FWMAVES are 228 mm in length, 22 mm in width, and 78 mm in height, with a total mass of 19.34 g, as summarized in Table 1. The basic structure of the MDD-FWMAVES uses a carbon frame. Two horizontal frames are connected via two vertical frames. Two motors also play a crucial role in supporting the two horizontal frames. The two wings are attached to two spur gears fixed to the horizontal frame, and two extension springs are connected between the vertical frames and the spur gears. The wings were fabricated using a modified KU beetle [14,35] and their planform area was enlarged compared to the previous version to increase the lift force. Each wing has a span of 83.82 mm and a mean chord length of 33.6 mm, resulting in a wing planform area of 2744.87 mm^2^. The communication board is mounted parallel to the vertical frame, and the control board is mounted parallel to the bottom of the horizontal frame. The control board, consisting of an MCU, IMU, and motor driver, controls the wing flapping.

The battery is mounted at the bottom of the MDD-FWMAVES to lower the center of gravity. The total mass of the vehicle was adjusted to approximately two-thirds of the maximum attainable thrust, ensuring a sufficient thrust margin for attitude control. To achieve this configuration, the mass of each manufactured component was directly measured using a high-resolution analytical balance and incorporated into the design. The resulting mass distribution is summarized in Table 1, where the arrangement was made symmetrically in both lateral and longitudinal directions so that the CG remained close to the geometric center of the vehicle.

**Figure 2:**
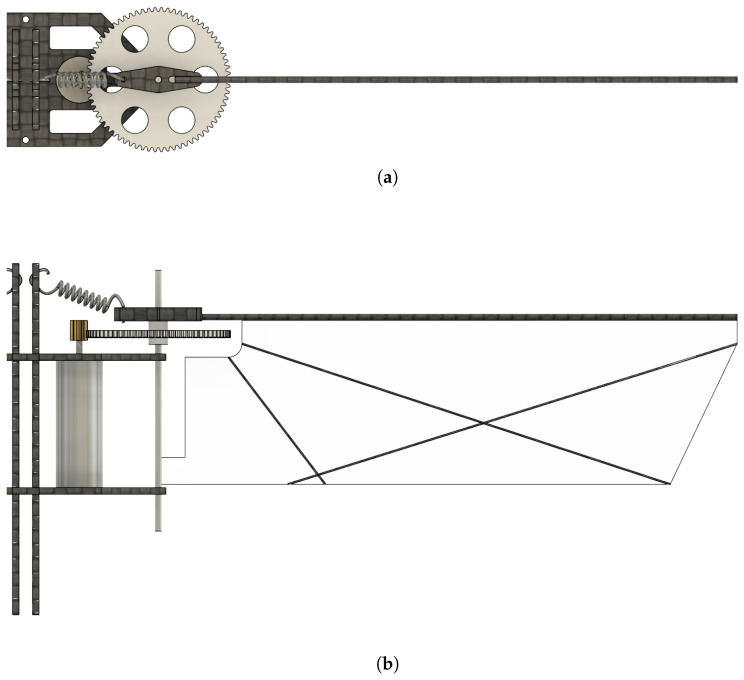
Schematics of one side of the flapping-wing actuation system with an extension spring: (**a**) Top view. (**b**) Front view.

Figure 2 shows the schematic diagram of the flapping-wing system of one wing of a MDD-FWMAVES. The flapping-wing system consists of a motor, pinion gear, spur gear, wing, wing cap, wing shaft, and extension spring. A pinion gear is mounted on the motor shaft and connected to a spur gear, which in turn is equipped with a wing cap that accommodates the wing and extension spring. The spur gear rotates in conjunction with the pinion gear, directly driving the wing flapping motion through the rotation of the motor.

One end of the extension spring is connected to the wing cap, and the other end is connected to the vertical frame. Conventionally, the springs are connected horizontally at the front and rear ends of the vertical frame of the MDD-FWMAVES to increase the spring extension distance [34]. While this method increases the spring extension distance and improves energy efficiency, the springs on both wings generate a coupled force, resulting in a relatively large yaw moment noise. To address this issue, the springs of the MDD-FWMAVES were connected to the center of the vertical frame to cancel out the yaw moment noise caused by both wings. Additionally, to ensure there is sufficient extension space for the extension spring, the attachment points of the vertical frame were installed diagonally higher than the wing cap.

### 2.2. Flapping-Wing Mechanism and Resonant Frequency

**Figure 3:**
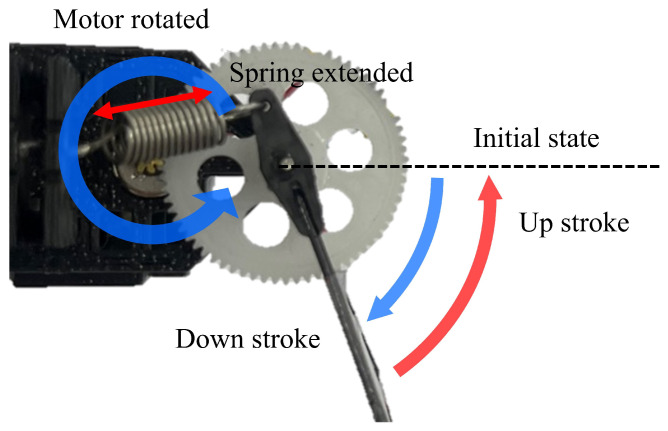
Flapping-wing actuation system mechanism with an extension spring.

Figure 3 illustrates the mechanism of a flapping-wing system that incorporates an extension spring. The direction and speed of the wing flapping are directly proportional to the rotational direction and speed of the motor. As the motor rotates, the pinion gear attached to it also rotates. This rotation causes the spur gear, which is connected to the pinion gear, to rotate in the opposite direction, which in turn moves the vanes connected to the spur gear via the wing cap. As the wing moves, the extension springs connected to the wing cap and the vertical frame are extended, storing energy. When the motor’s rotational direction reverses, the stored energy assists the motion, thereby increasing energy efficiency.

Flapping can be viewed as a repetitive up-and-down motion of the wing, and flapping can be performed repeatedly by rapidly changing the motor’s rotational direction. As the flapping motion is repeated, the extension spring also repeatedly contracts and expands. The extension spring applies force in the opposite direction to the flapping direction and accumulates elastic energy as its length increases. When the wing reaches the end point of the upstroke or downstroke, the extension spring is fully extended. At the endpoint, the motor’s rotational direction reverses, resulting in continued flapping. Because the motor must apply torque in the opposite direction of its current motion, a high torque is necessary to overcome the inertia moment. The elastic energy stored in the extension spring while the wing moves to one end is used to change the wing’s direction at the end position of the wing motion, reducing the energy required by the motor. The flapping system equations for a MDD-FWMAVES were derived from a previous study [34]. The opposite torque τ generated by the extension spring when the wing rotates by $\theta_{S}$ from the center is given as follows:

(1) $$\tau =\left(n\left(I_{M}+I_{P}\right)+\frac{1}{n}\left(I_{S}+I_{W}\right)\right){\ddot{\theta }}_{S}+\left(n\left(D_{M}+D_{P}\right)+\frac{1}{n}\left(D_{S}+D_{W}\right)\right){\dot{\theta }}_{S}+\left(\frac{k_{r}}{n}\right)\theta_{S}.$$

Here, $n=\frac{n_{s}}{n_{p}}$ corresponds to the gear ratio; $n_{s}$ and $n_{p}$ are the number of teeth of the spur gear and the number of teeth of the pinion gear, respectively. $I_{M},\;I_{P},\;I_{S}$, and $I_{W}$ are the moments of inertia of the motor, pinion gear, spur gear, and wing, respectively. $D_{M}$ and $D_{P}$ represent the damping coefficients of the motor and that of pinion gear, respectively. $D_{S}$ and $D_{W}$ are the linearly approximated damping coefficients of the spur gear and that of the wing, respectively. $k_{r}$ is the rotational spring coefficient of the extension spring and the total spring constant $\left(\frac{k_{r}}{n}\right)$ as $K_{T}$.

The above equation is a second-order differential equation for $\theta_{S}$. When the coefficient of the second-order term is defined as the total moment of inertia $I_{T}$, i.e., $I_{T}:=\;n\left(I_{M}+I_{P}\right)+\frac{1}{n}\left(I_{S}+I_{W}\right)$, the resonant frequency $f_{n}$ of the entire system is calculated as follows:

(2) $$f_{n}=\frac{\omega_{n}}{2\pi }=\frac{1}{2\pi }\sqrt{\frac{K_{T}}{I_{T}}}=\frac{1}{2\pi }\sqrt{\frac{k_{r}}{n}\cdot \frac{1}{I_{T}}}$$

Among these, $I_{T}$ is the value based on the inertias of the motor, pinion gear, spur gear, and wing, and it can be calculated using CAD programs. The spring constant term can be derived by calculating the torque generated by the extension spring during wing flapping motion and deriving the rotational spring constant.

However, the equation based on the above model may differ from the actual resonant frequency due to theoretical calculations of the moment of inertia and linearization in the rotational spring constant calculation. Therefore, the actual resonant frequency was measured experimentally to validate the theoretically derived value. The resonant frequency can be measured as the frequency at which the wing flapping amplitude is greatest when constant motor thrust is applied and the frequency is varied.

**Figure 4:**
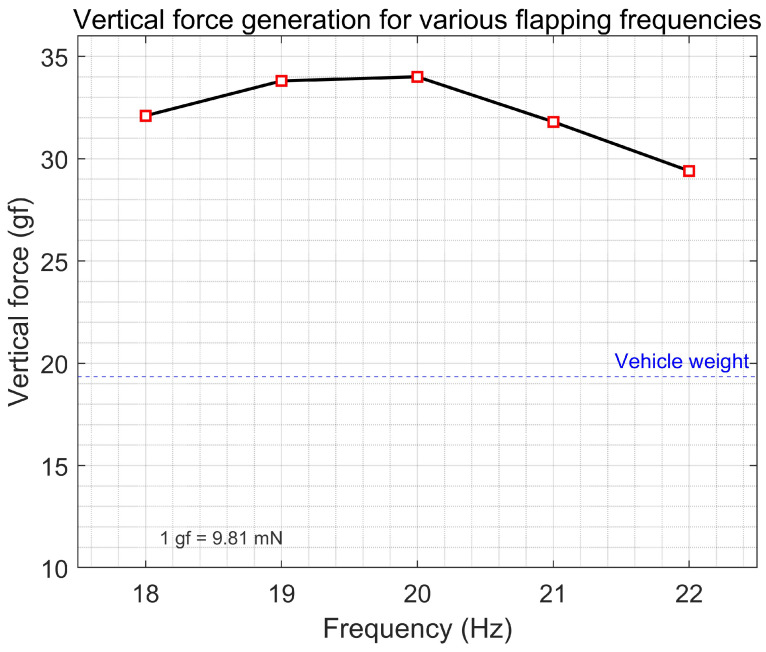
Vertical force generation for various flapping frequencies.

Table 2 shows the values of each parameter. In actual systems, deviations may occur due to inaccurate moments of inertia modeling and spring linearization. Therefore, under the condition of maintaining the same flapping amplitude, the driving frequency was swept across the 18–22 Hz range, and the thrust was measured using a six-axis load cell (Nano17, ATI Industrial Automation, Apex, NC, USA, force resolution ≈0.3gf, torque resolution ≈0.0156Nmm). The maximum thrust response was observed at 20 Hz, and thus 20 Hz was selected as the operating frequency, as shown in Figure 4.

## 3. Control System Design

This section presents the design of the attitude controller for the MDD-FWMAVES system. First, we describe the control inputs for roll, pitch, and yaw attitude control, and we present the thrust and moment measurements. We then describe the design of an IMU filter for attitude estimation as well as the design and verification of a PD controller.

### 3.1. Flapping-Wing Control Mechanism

**Figure 5:**
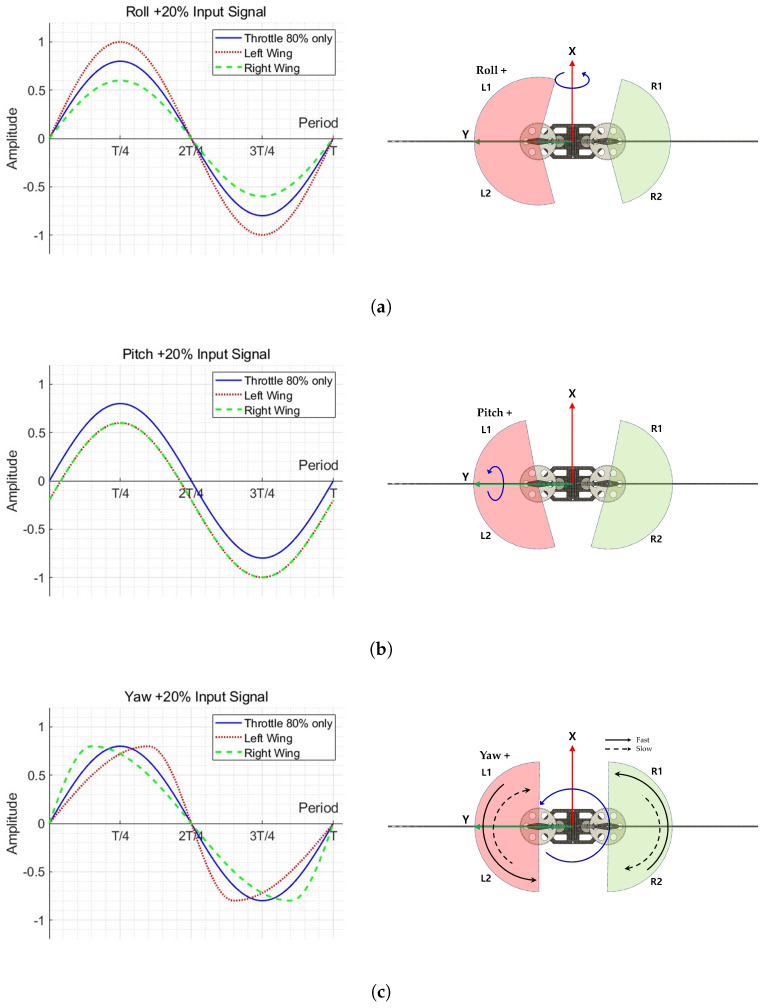
Driving signal waveforms and wing flapping variation for attitude controls: (**a**) Roll. (**b**) Pitch. (**c**) Yaw.

Figure 5 shows the waveform of the drive signal for the attitude control of the MDD-FWMAVES and the corresponding change in wing flapping [34]. Since the wing position is proportional to the integral of the motor’s rotational speed, and the motor rotational speed is controlled by the drive signal, the wing position is proportional to the integral of the drive signal. The MDD-FWMAVES with a direct motor drive generates wing flapping by periodically changing the rotational direction of the motor using a sinusoidal drive signal. The motor is driven through an H-bridge circuit, and the rotational speed is controlled by the duty cycle of the PWM signal applied to the H-bridge. The PWM frequency was 1 kHz. The 20 Hz sine wave was used for the motor drive signal to utilize the resonance effect. In Figure 5, the blue line represents the basic drive signal at 80% thrust, while the dotted and dashed lines represent the modified drive signals used to generate roll, pitch, and yaw moments for the left and right wings, respectively. The diagram on the right shows the wing flapping drive planes corresponding to the signals. Roll, pitch, and yaw moments are generated by asymmetrically adjusting sine waves. The roll moment is generated by differentiating the sine wave amplitudes to account for the difference in wing flapping amplitudes between the left and right wings. The graph in Figure 5a shows the signal graphs for the left and right wings when generating a roll-right moment. The signal entering the left wing increases the amplitude of the basic drive signal. Conversely, the signal entering the right wing decreases the amplitude, generating more thrust on the left wing than on the right wing, resulting in a roll-right moment. The pitch moment is generated by offsetting the sine wave to shift the center of the wing flapping motion forward or backward. The graph in Figure 5b shows the signals input to the left and right wings when generating a pitch-down moment. Both signals input to the left and right wings decrease the offset from the basic drive signal, increasing the rear wing flapping and reducing the front wing flapping. This generates more thrust at the rear of the MDD-FWMAVES than at the front, generating the pitch-down moment. The yaw moment is generated by differentiating the ascent and descent speeds of the sine waves, exploiting the difference in the upstroke and downstroke flapping speeds of the two wings. This is implemented using a split-cycle method [36,37]. Figure 5c shows the signal that generates a counterclockwise yaw moment on the left and right wings. For the left wing, the period increases during ascent and decreases during descent, resulting in a slower upstroke and a faster downstroke, creating a speed difference. The right wing, on the other hand, has a faster upstroke and a slower downstroke, generating asymmetric flapping between the two wings to generate the yaw moment.

**Figure 6:**
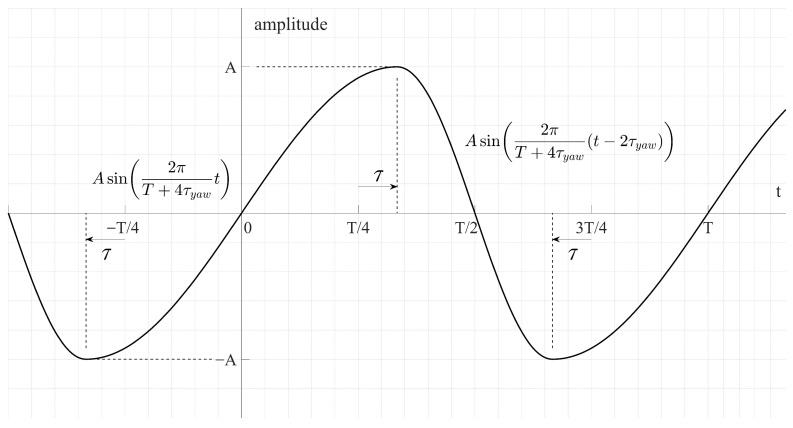
Sine wave input signal for yaw.

Figure 6 shows a yaw drive signal by split-cycle. The period *T* of the drive signal is the same as the original sine wave, but when rising, it is applied as a sine wave with a 1/4 period increased by τ, and when falling, it is applied as a sine wave with a 1/4 period decreased by τ.

Considering all roll, pitch, and yaw drive inputs, in the first cycle (n=0), the drive inputs for the left and right motors are shown bellow. In the table, the original sine wave is $A\sin\frac{2\pi }{T}t$. $A_{\mathrm{roll}}$ represents the roll drive input, $B_{\mathrm{pitch}}$ represents the pitch drive input, and $\tau_{\mathrm{yaw}}$ represents the yaw drive input. In subsequent cycles, the signals in Table 3 are repeated.

### 3.2. Measurement of Forces and Moments

**Figure 7:**
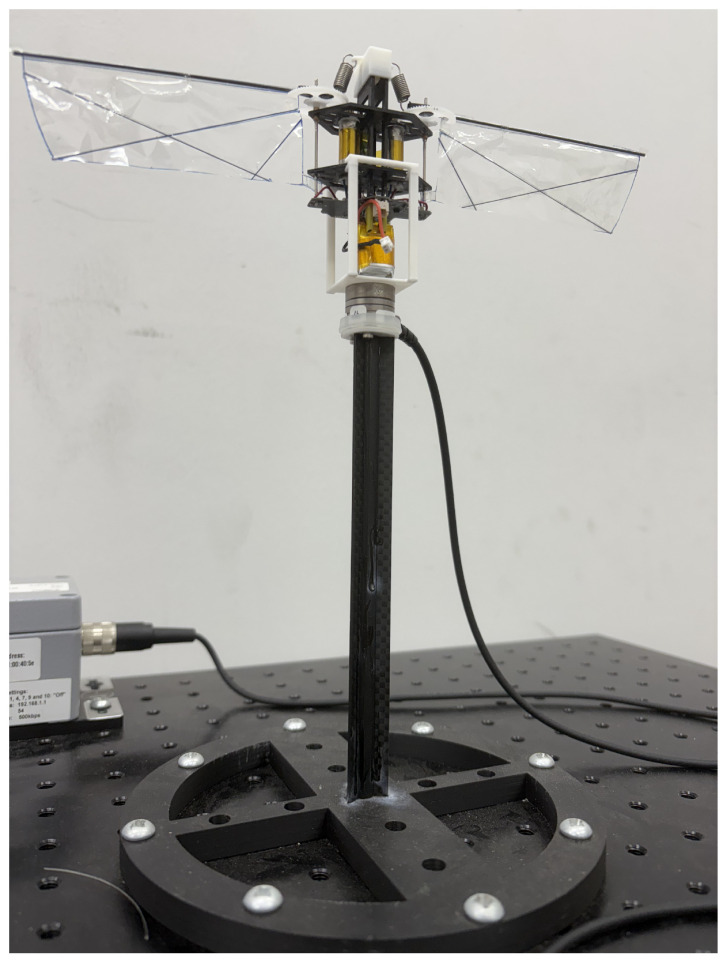
Experimental setup for force and torque measurement using the load cell Nano 17.

For the MDD-FWMAVES to fly stably, it must generate sufficient thrust and moments along the roll, pitch, and yaw axes. A six-axis load cell (Nano17, ATI Industrial Automation, USA, force resolution ≈0.3gf, torque resolution ≈0.0156Nmm) was used to measure thrust and moments. The experimental setup is presented in Figure 7. The MDD-FWMAVES was first secured to a 3D-printed support frame, which was then mounted on top of the load cell. The load cell was then mounted on a carbon fiber pillar frame, positioned at the same height as the wingspan to reduce ground effect. In this configuration, MDD-FWMAVES was mounted near the center of gravity (CG) in the load cell’s measurement coordinate system, so the measured torque can be considered a value obtained with respect to the CG. During the experiment, power was supplied by an external DC power supply (7.8 V) instead of the onboard battery, which simulates approximately 60% charge conditions of a two-cell 7.4 V Li-Po battery.

**Figure 8:**
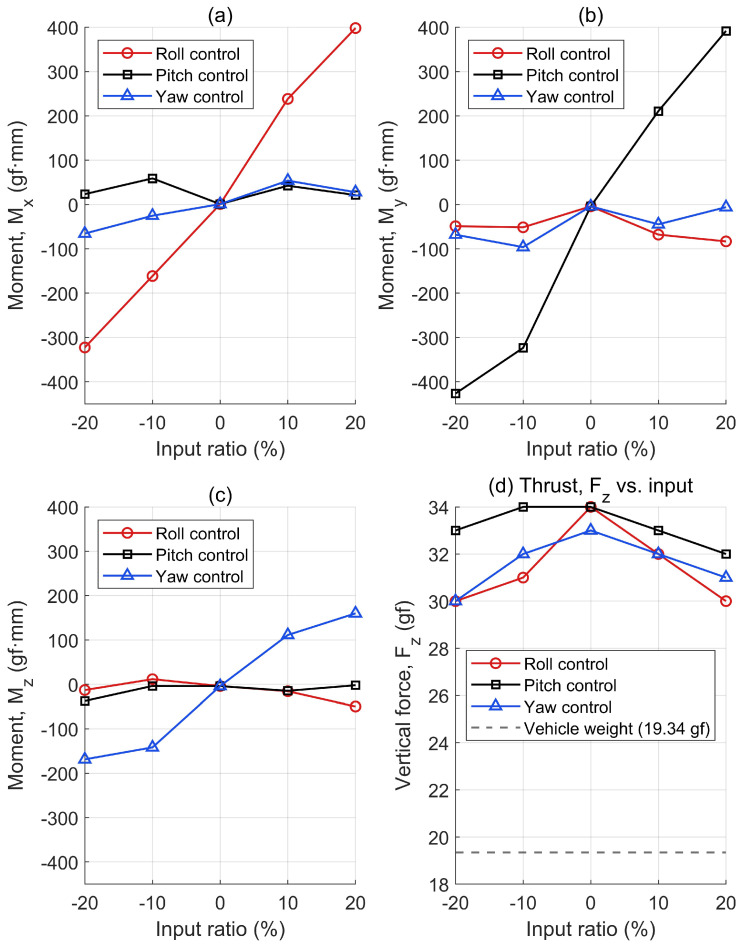
Generation of forces and moments with different wing motions: (**a**) Roll moment. (**b**) Pitch moment. (**c**) Yaw moment. (**d**) Vertical force.

Figure 8 shows the roll, pitch, yaw moment, and thrust measurements of the MDD-FWMAVES. The axial moment and thrust of the MDD-FWMAVES were measured using the method described in Figure 5 by applying a control input at a resonant frequency of 20 Hz and varying the roll, pitch, and yaw control inputs from −20% to +20%.

Figure 8a shows that changing the control input for roll control from −20% to +20% generates a roll moment varying from approximately −322 to 398 gf·mm. It shows that almost no roll moment is generated by the pitch and yaw inputs.

Similarly, Figure 8b shows that changing the control input for pitch control from −20% to +20% generates a linear pitch moment varying from approximately −426 to 391 gf·mm. It also shows that almost no pitch moment is generated by the roll input and yaw input.

Figure 8c shows that the yaw moment varies linearly with the yaw control input. However, the yaw moment for the yaw control input is approximately half the size of the roll moment for the roll input and the pitch moment for the pitch input. This is because the roll and pitch moments are generated by asymmetrical vertical forces, while the yaw moment is generated by asymmetrical horizontal forces, making moment generation difficult. Additionally, it can be seen that the yaw moment is hardly generated by the roll input and pitch input.

The vertical force in the z-direction remained around 30 g, even when the roll, pitch, and yaw inputs were varied. At a charge condition of approximately 60%, the generated thrust was about 55% greater than the MDD-FWMAVES’s mass of 19.34 g, providing sufficient lift to fly the vehicle.

### 3.3. Attitude Estimation and Filter Design

**Figure 9:**
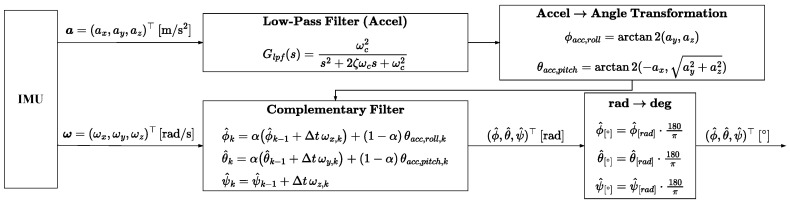
Block diagram of the IMU filter.

To accurately estimate a MDD-FWMAVES’s attitude, the angular velocity and acceleration data measured via the Inertial Measurement Unit (IMU) are used to calculate the roll, pitch, and yaw angles. Since the IMU is included in the control board and moves with the MDD-FWMAVES, vibration and noise caused by the wing flapping can affect the measured data. Therefore, it is essential to design a filter that effectively removes this noise and vibration. In this study, as shown in Figure 9, appropriate filters were designed for each accelerometer and gyroscope signal to accurately estimate the attitude angles.

Figure 9 shows the IMU-based attitude estimation pipeline. The acceleration measured by the IMU is output as $a={\left(a_{x},a_{y},a_{z}\right)}^{⊤}$, and the gyroscope angular velocity is output as $\omega ={\left(\omega_{x},\omega_{y},\omega_{z}\right)}^{⊤}$.

A second-order low-pass filter (LPF) is first applied to the acceleration output to suppress vibration and high-frequency noise. In this study, the cutoff frequency was set to $\omega_{c}=31.416\;\mathrm{rad}/s$. Afterwards, using the transformation formulas presented in “Accel → Angle Transformation” in Figure 9, the roll ($\phi_{\mathrm{acc}}$) and pitch ($\theta_{\mathrm{acc}}$) were calculated based on the gravity vector.

While these estimates are stable in the low-frequency range, they can deviate in the dynamic range due to the influence of linear acceleration.

The gyro output rapidly reflects attitude changes by integrating the angular velocity $\left(\omega_{x},\omega_{y},\omega_{z}\right)$ over time. The gyro integration value is fast and robust to dynamic movements, but drift occurs during long-term operation due to accumulated bias and measurement noises.

The “Complementary Filter” in the block diagram combines the acceleration-based angle and the gyro-integrated angle, balancing the strengths and weaknesses of these two sensor outputs. The combined weight is controlled by the complementary filter constant α. A larger α value assigns more weight to the gyro component. In this study, $\alpha_{\phi }$ was set to 0.96 for roll and $\alpha_{\theta }$ was set to 0.80 for pitch. The complementary filter output was calculated in radians (rad) and converted to degrees in the “rad → deg” conversion block in the block diagram. The final result, ${\left(\hat{\phi },\hat{\theta },\hat{\psi }\right)}^{⊤}$, is given in degrees (°).

Since the yaw angle is calculated only by integrating the gyroscope data without applying the complementary filter, the accuracy of the estimated value depends on the accuracy of the gyroscope data.

### 3.4. PD Control Theory and Hardware

Active feedback control must be considered to stabilize a tailless FWMAV during flight. Among various control methods, a simple PD controller has demonstrated stable flight in several FWMAVs, including Robobee [37], Delfly [19], Hummingbird Robot [32] and KU beetle [14], by sensing three Euler angles.

**Figure 10:**
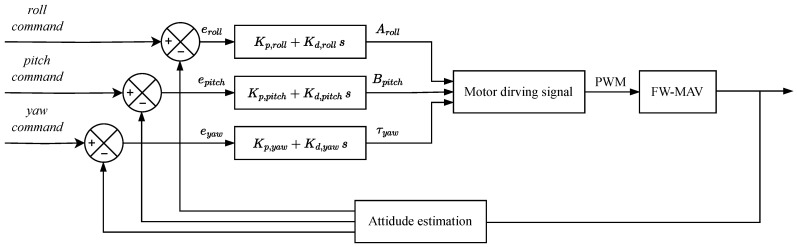
Block diagram of the PD control system.

In this study, a PD controller was applied to the attitude control of the MDD-FWMAVES. Figure 10 illustrates the attitude controller of the MDD-FWMAVES. Reference values for roll, pitch, and yaw are provided by the RF transmitter, and the MDD-FWMAVES’s attitude values are estimated utilizing the measured data by IMU. The errors between the reference values and the estimated attitude values are then used as inputs to perform PD control for each of the following three control axes: roll, pitch, and yaw.

From the PD control results, the control parameter values $A_{\mathrm{roll}}$, $B_{\mathrm{pitch}}$, and $\tau_{\mathrm{yaw}}$ in Table 3 were determined. The drive signal was converted to PWM to drive the motors for each wing flapping. The proportional gain ($K_{P}$) and differential gain ($K_{D}$) values of the PD controller were established through flight experiments.

### 3.5. Attitude Control Using a Gimbal System

**Figure 11:**
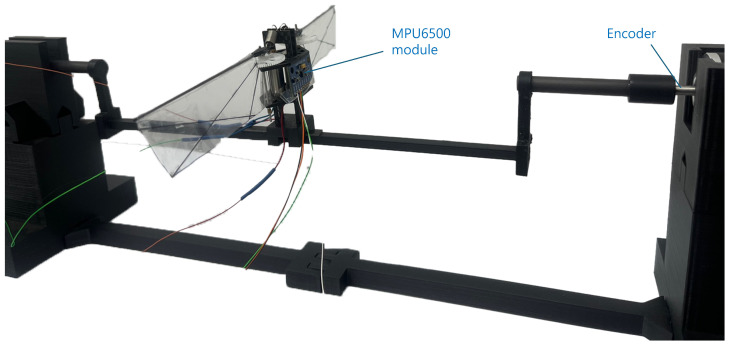
Single-axis gimbal and MDD-FWMAVES for the attitude control test.

To verify the accuracy of onboard sensing and controllability along the roll and pitch axes, angular control experiments of the MDD-FWMAVES were conducted using a single-axis gimbal. As shown in Figure 11, the single-axis gimbal was designed so that its rotation axis passes through the center of gravity of the MDD-FWMAVES and incorporates a central groove to fix the vehicle for either roll or pitch testing. An encoder with a resolution of $0.003^{\circ }$ was mounted on one end of the gimbal shaft, enabling the precise measurement of angular displacements. The gimbal assembly has overall dimensions of 450 mm × 100 mm × 120 mm. During the experiments, the drive power was supplied from a DC power supply at 7.8 V, which corresponds to the 60% charge state of a two-cell 7.4 V Li-Po battery. The throttle input was fixed at approximately 80% of the maximum thrust, and each axis was controlled by a PD controller, as described in the previous section. Figure 10 shows the attitude angle responses measured by the encoder when step inputs of $0^{\circ }$, $10^{\circ }$, and $20^{\circ }$ were applied to the roll and pitch axes. It was confirmed that the PD controller quickly followed the target angle in both the roll and pitch axes. These results demonstrate that the attitude of the MDD-FWMAVES is well estimated, while the PD controller effectively performs angle control.

**Figure 12:**
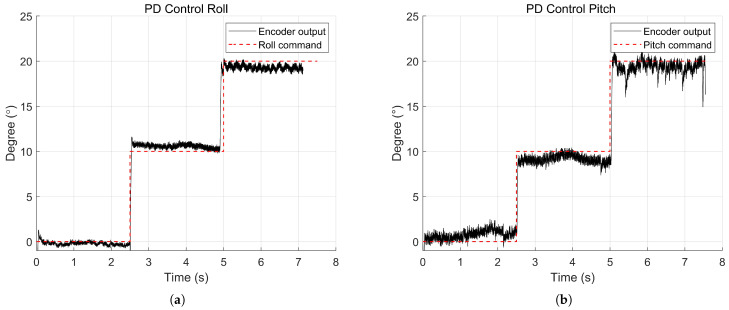
PD controller experimental results for the MDD-FWMAVES using a single-axis gimbal: (**a**) Roll. (**b**) Pitch.

Figure 12 shows the attitude angle responses measured by the encoder when step inputs of $0^{\circ }$, $10^{\circ }$, and $20^{\circ }$ were applied to the roll and pitch axes. It was confirmed that the PD controller quickly followed the target angle in both the roll and pitch axes. These results demonstrate that the attitude of the MDD-FWMAVES is well estimated while the PD controller effectively performs angle control, and they verify that sufficient roll and pitch control forces are generated to track the commanded angles.

Due to the weight of the gimbal system, etc., the control gains obtained while stabilizing the MDD-FWMAVES using the gimbal system cannot be directly used for free flight control. However, experiments using the gimbal system have served as a useful reference for reducing the number of flight tests required to determine the appropriate control gains.

## 4. Flight Experiment

### 4.1. Onboard Control System Design

**Figure 13:**
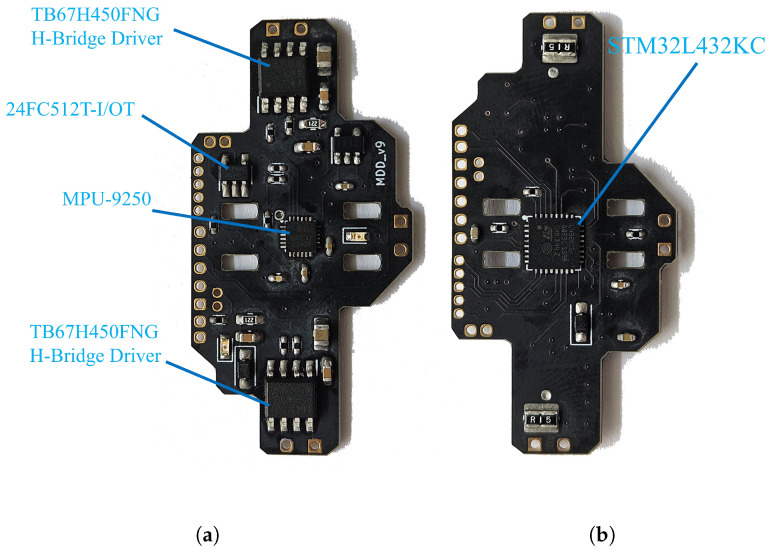
Control board for the MDD-FWMAVES: (**a**) Top. (**b**) Bottom.

To evaluate the performance of the attitude control mechanism of the MDD-FWMAVES, free flight experiments were conducted. The MDD-FWMAVES was powered by a two-cell series-connected 7.4 V lithium-polymer (Li-Po) battery. For the onboard attitude control experiments, an integrated control board (Figure 13) was designed.

Table 4 contains detailed specifications of the control board. The control board consists of an MCU, IMU, motor driver, and EEPROM.

**Figure 14:**
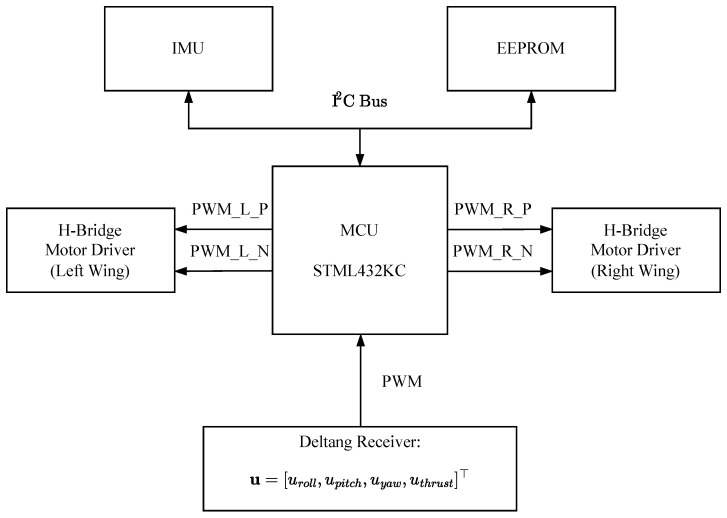
Signal flow diagram of the control board.

The signal flow of the control board is shown in Figure 14. The MCU generates control signals using attitude data received from the IMU and input from the Deltang RC receiver. These control signals are converted into PWM signals and then transmitted to the motor drivers. The attitude data and control input signals from the MCU during flight are stored to the EEPROM. The control board and motor driver were supplied with 7.4 V, while the MCU, IMU, EEPROM, and DelTang receiver were supplied with 3.3 V through the onboard regulator. Thrust was manually adjusted using the throttle stick of the remote controller.

### 4.2. Flight Experiment Result

The flight experiments were conducted to demonstrate the hovering capability of the MDD-FWMAVES. For this purpose, the reference angles of the roll and pitch PD controllers were set to 0°, and only the throttle input was varied during the experiments without additional external control factors. This configuration was chosen to evaluate the feasibility of stable hovering under minimal control conditions.

The control gains obtained from the preliminary gimbal-based tests were used as an initial reference. While the gimbal setup provided a useful basis for PD control tuning, the gains required further adjustment because the free flight environment differed significantly from the constrained gimbal conditions. The final control gains were therefore determined through iterative free flight experiments.

**Figure 15:**
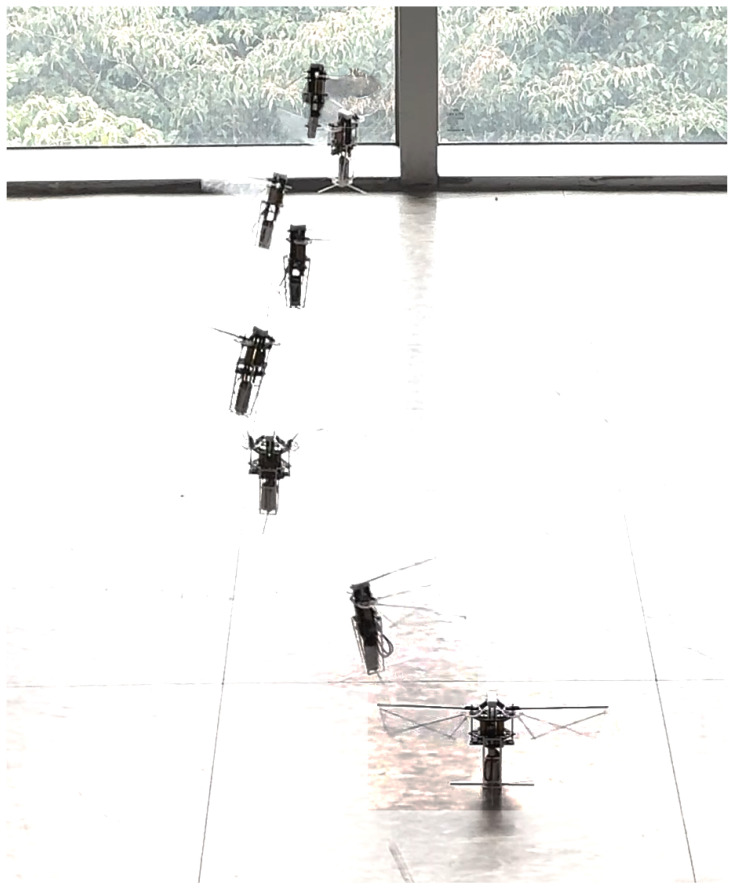
Composite images of the MDD-FWMAVES captured by a camera.

During these tests, video recordings were captured at 60 fps using a camera, which provided visual confirmation of the hovering stability and were used to qualitatively assess the vehicle’s attitude response. In parallel, the MDD-FWMAVES attitude data (roll, pitch, and yaw angles) and control inputs (throttle and PD controller outputs) were simultaneously logged in the onboard EEPROM at 10 Hz, enabling the quantitative analysis of the system’s response. Representative snapshots of the MDD-FWMAVES in flight are presented in Figure 15, where stable hovering flight was successfully demonstrated for approximately 6 s. These results indicate that the roll and pitch control forces validated in the gimbal experiments were sufficient to achieve stable hovering in free flight, as the vehicle maintained its position within about ±10 cm using only onboard sensing and control.

**Figure 16:**
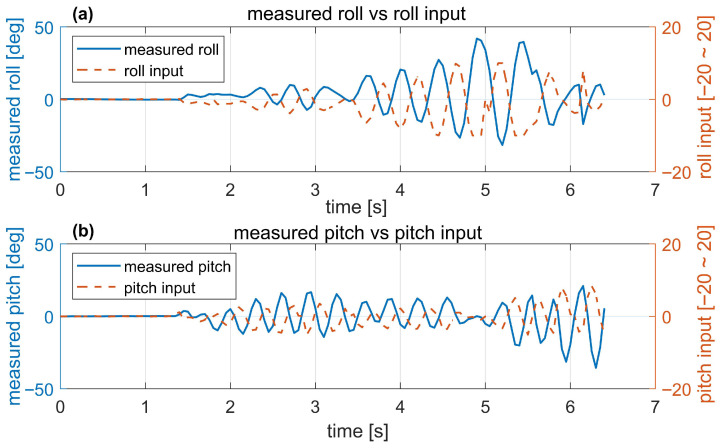
Measured roll and pitch angles recorded during free flight. (**a**) Roll. (**b**) Pitch.

Attitude data acquired via the IMU during flight are shown in Figure 16. In Figure 16a, the solid line is the real roll angle measured during free flight, and the dashed line shows the control input by the roll controller. In Figure 16b, the solid line is the real pitch angle measured during free flight, and the dashed line is the control input by the pitch controller to keep the hovering condition. It can be seen that the pitch and roll angles exhibit some oscillation around the reference angle of 0°, but remain stable overall.

The phase lead of the roll and pitch control inputs observed in Figure 16 occurs because the derivative term of the PD controller directly uses the gyroscope angular velocity for fast control response, while the measured attitudes are delayed by sensor fusion and low-pass filtering.

The yaw control is currently being tested to overcome vibration and is not currently in use. Although yaw control was not implemented, roll and pitch were observed to remain stable during flight. However, because yaw control was not available, it was difficult to fly the MDD-FWMAVES to the desired destination using the RF remote controller. To increase flight time and ensure practical use, yaw control is necessary.

## 5. Conclusions

This study presents the design and stable flight of the insect-like MDD-FWMAVES. This robot uses directly driven motors and extension springs to achieve wing flapping. Individual motors are used for each wing, enabling attitude control through individual wing flaps without the need for additional servo motors. Series of experimental tests have demonstrated that individual wing flaps can generate the desired roll, pitch, and yaw moments. An onboard control system was designed and tested, demonstrating successful free flight with very small changes in the roll and pitch behavior of the MDD-FWMAVES. Currently, only roll and pitch control is implemented, and a yaw controller should be added for practical applications. Furthermore, while roll and pitch control has been stabilized to a certain degree through trial-and-error methods, model-based controller design is necessary to further improve flight performance.

## Figures and Tables

**Table 1:** Weight composition of the MDD-FWMAVES model.

Component	Weight (g)	Quantity	Sub Total Weight (g)
Motor	3.55	2	7.1
Spring	0.31	2	0.62
Wing shaft	0.21	2	0.42
Wing cap	0.1	2	0.2
Spur gear	0.36	2	0.72
Pinion gear	0.05	2	0.1
Wing	0.06	2	0.12
Frame	2.6	1	2.6
Control board	1.5	1	1.5
RF receiver	0.41	1	0.41
Battery	4.8	1	4.8
Etc.	-	-	0.75
Total weight (g)			19.34

**Table 2:** Parameters of the MDD-FWMAVES model.

Parameter	Description	MDD-FWMAVES Model Value
$I_{T}$	Total moment of inertia	$7.25\times 10^{-8}\;\mathrm{kg}\cdot m^{2}$
$k_{e}$	Extension spring constant	0.490N/mm
$k_{r}$	Rotational spring constant	0.0110N·m/rad
*n*	Gear ratio	70:7
$f_{n}$	Natural frequency	19.59Hz
*w*	Total weight	19.34g

**Table 3:** Control signals for left and right motors.

	$-T/4-\tau_{\mathrm{yaw}}\leq t\leq T/4+\tau_{\mathrm{yaw}}$ (Up-Stroke)	$T/4+\tau_{\mathrm{yaw}}\leq t\leq 3T/4-\tau_{\mathrm{yaw}}$ (Down-Stroke)
Left motor	$\left(A+A_{\mathrm{roll}}\right)\sin\;\left(\frac{2\pi }{T+4\tau_{\mathrm{yaw}}}\;t\right)+B_{\mathrm{pitch}}$	$\left(A+A_{\mathrm{roll}}\right)\sin\;\left(\frac{2\pi }{T-4\tau_{\mathrm{yaw}}}\;\left(t-2\tau_{\mathrm{yaw}}\right)\right)+B_{\mathrm{pitch}}$
Right motor	$\left(A-A_{\mathrm{roll}}\right)\sin\;\left(\frac{2\pi }{T-4\tau_{\mathrm{yaw}}}\;t\right)+B_{\mathrm{pitch}}$	$\left(A-A_{\mathrm{roll}}\right)\sin\;\left(\frac{2\pi }{T+4\tau_{\mathrm{yaw}}}\;\left(t+2\tau_{\mathrm{yaw}}\right)\right)+B_{\mathrm{pitch}}$

**Table 4:** Specification of components of the MDD-FWMAVES.

Component	Specification
MCU	STM32L432KC
Motor driver	TB67H450FNG H-bridge driver
IMU	MPU-9250
EEPROM	24FC512T-I/OT
Communication	Deltang DT-RX35d DSM2-compatible receiver
Battery	7.4 V 2-cell Li-Po

## Data Availability

The experimental data supporting the findings of this study are publicly available in the Zenodo repository at https://doi.org/10.5281/zenodo.17104857.
